# Assessment of clinicopathologic features in patients with pituitary adenomas in Northeast of Iran: A 13-year retrospective study

**Published:** 2015-10-07

**Authors:** Kazem Anvari, Mahmoud Reza Kalantari, Fariborz Samini, Soodabeh Shahidsales, Mehdi Seilanian-Toussi, Zakiyeh Ghorbanpour

**Affiliations:** 1 Cancer Research Center AND Department of Radiation Oncology, School of Medicine, Mashhad University of Medical Sciences, Mashhad, Iran; 2 Department of Pathology, School of Medicine, Ghaem Hospital, Mashhad University of Medical Sciences, Mashhad, Iran; 3 Department of Neurosurgery, School of Medicine, Shahid Kamiab Hospital, Mashhad University of Medical Sciences, Mashhad, Iran; 4 Department of Radiation Oncology, School of Medicine, Omid Hospital, Mashhad University of Medical Sciences, Mashhad, Iran

**Keywords:** Pituitary Adenoma, Functional Adenoma, Survival Rate

## Abstract

**Background:** Intracranial lesions of the pituitary gland are common pituitary adenomas, accounting for 6-10% of all symptomatic intracranial tumors. In this retrospective study, the clinicopathologic features and survival rate of pituitary adenomas were evaluated.

**Methods: **The present retrospective study was conducted on 83 patients with pituitary adenomas, referring to radiation oncology departments of Ghaem and Omid Hospitals, Mashhad, Iran, over a period of 13 years (1999-2012). Data obtained from clinical records including clinical features, type of surgery (if performed), treatment modality, overall survival rate, and progression-free survival rate were analyzed.

**Results: **Eighty-three patients including 44 males (53%) and 39 females (47%) participated in this study. The median age was 40 years (age range: 10-69 years). Chiasm compression was reported in 62 patients (74.4%), and 45.78% of the subjects suffered from headaches. Functional and non-functional adenomas were reported in 44 (53.01%) and 39 (46.99%) patients, respectively. In cases with functional and non-functional adenomas, the disease was controlled in 95 and 84.5% of the subjects for 3 years, respectively. Furthermore, 1- and 3-year survival rates for functional adenoma were 84.6 and 23%, respectively; the corresponding values were 90.9 and 22.7% in non-functional adenomas, respectively.

**Conclusion:** In this study, a significant correlation between headache severity and type of adenoma was observed. So, application of surgery and radiotherapy together could be a highly effective approach for treating functional adenomas, although it is less efficient for the non-functional type.

## Introduction

Intracranial lesions of pituitary gland are common pituitary adenomas, accounting for 6-10% of all symptomatic intracranial tumors.^1^^-^^3^ Pituitary adenomas are defined as abnormal growth of tumors in pituitary glands (benign adenomas, invasive adenomas, and carcinomas).^4^^,^^5^ Recent studies have shown that invasive adenomas may approximately affect 1 in 1000 people of the general population.^6^

The most frequent pituitary adenomas are microadenomas with an estimated incidence of 16.7%. Pituitary adenomas are also categorized as active-functioning and non-functioning adenomas; two-thirds of clinically diagnosed lesions are functional adenomas.^7^

Symptoms of pituitary disorders are often non-specific and may differ given the effects of space-occupying lesions, increased hormonal release or both.^8^^,^^9^

Among patients with pituitary adenomas, different types of headaches such as chronic, episodic migraines and unilateral headaches including primary stabbing headache, short-lasting unilateral neuralgiform headache, cluster headache, and hemicrania continua are common.^10^^-^^13^ Pituitary adenomas are also associated with psychiatric disorders including hostility, anxiety, apathy, depression, emotional instability, and irritability.^14^^,^^15^

Although the treatment of pituitary adenoma depends on the size and type of tumor, surgery is the common treatment modality. Transsphenoidal adenomectomy is a method for tumor removal, though recently, endoscopic surgery has been commonly applied.^16^ Due to the importance of pituitary disorders and insufficient research in this field, this study aimed to assess the clinicopathologic features and treatment outcomes of patients with pituitary adenomas over a 13-year period in the Northeast of Iran.

## Materials and Methods

We studied the clinicopathologic features of all patients, presenting with pituitary adenomas. The subjects had referred to the departments of radiation oncology at Omid and Ghaem hospitals in years 1999-2012.

The sample size included 83 patients, according to inclusion criteria. Since all eligible subjects were recruited within a specific time span, use of a formula for calculating the sample size was not necessary. The inclusion criteria were as follows: (1) Pathological evidence of pituitary adenoma; (2) essential information including age, gender, treatment modality, and type of surgery and medical records; and (3) undergoing medically proposed treatments. The exclusion criterion was unfinished complementary treatment (the recommended treatment).

In this retrospective, cohort study, all patients’ medical records were collected and examined after review and pathological assessment for the selection of definitive or complementary treatments. Data obtained from the clinical records such as clinical signs, type of surgery (if performed), treatment modality, overall survival rate, and progression-free survival were examined; in addition, previous medical histories and clinical variables were recorded. We contacted the patients in case the data needed to be corrected or completed.

Patients with pituitary adenomas, diagnosed via pathological assessment were included in this study after meeting the inclusion criteria. All patients’ records were included in the predesigned questionnaires.

Data obtained from patients’ records and recorded calls were analyzed by SPSS software (version 16, SPSS Inc., Chicago, IL, USA). For descriptive data, statistical indices, tables, and diagrams were used. Conventional methods of survival analysis including Kaplan–Meier and Cox regression were employed in order to study the effects of variables on disease-free survival rate.

Since no medical interventions were performed in this study and the patients’ records were kept confidential, no written consents were obtained.

## Results

Eighty-three patients including 44 males (53%) and 39 females (47%) participated in this study. 4, 12, 24 and 19 patients were within the age range of 10-20, 20-29, 30-39, and 40-49 years, respectively; in addition, 19 patients were 50-59 years old, and five subjects were within the age range of 60-69 years. The median age was 40 years (age range: 10-69 years), and the majority of the subjects (28.9%) were 30-39 years old.

Chiasm compression was reported in 62 patients (74.4%) and 45.78% of the subjects suffered from headaches. Functional and non-functional adenomas were observed in 44 (53.01%) and 39 (46.99%) patients, respectively.

**Figure 1:**
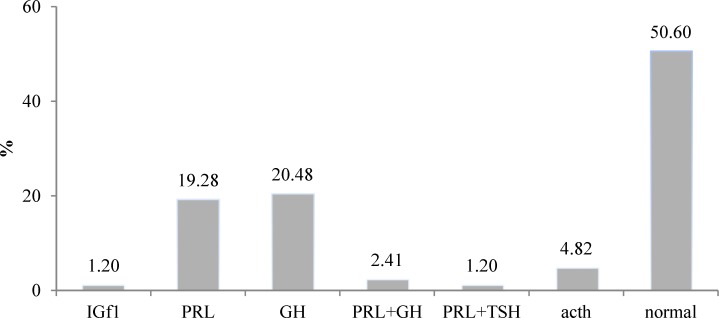
Increased secreted hormones

IGF1: Insulin-like growth factor-1; PRL: Prolactin; GH: Growth hormone; TSH: Thyroid stimulating hormone

Prolactin (PRL) and growth hormones (GH) were the most secreted hormones (19.3% and 20.5%, respectively). Of all patients, one showed an increased insulin-like growth factor-1 (IGF-1) level, 16 experienced prolactin elevation and 17 cases had elevated growth hormone; in addition, adrenocorticotropic hormone (ACTH) level increased in 4 cases (Figure 1).

Galactorrhea and acromegaly were observed in 6% and 22% of the patients, respectively, and Cushing’s syndrome was reported in 4.82% of the subjects; this disease recurred in 25 patients (30%).

Among 44 patients (53%) with functional adenomas, 22 subjects were males (50%) and 22 were females (50%). Thirty-nine (47%) individuals suffered from non-functional adenomas, including 22 males (50%) and 17 females (43.6%). According to the findings of the present study, no significant association was found between adenoma type and gender (P = 0.540).

Among 44 patients with functional adenomas, 25 patients aged < 40 years (56.8%) and 19 cases were > 40 years (43.2%). Furthermore, in patients with non-functional adenomas, 17 and 22 individuals were < 40 and > 40 years of age, respectively (43.6% and 56.4%, respectively). There was no significant association between age and type of adenoma (P = 0.229).

Chiasm compression, accompanied by adenoma, was assessed in patients with functional and non-functional adenomas. Signs of compression were reported in 31 patients with functional adenomas (70.5%) and 31 subjects with non-functional adenomas (79.5%); no significant relationship was observed between chiasm compression and tumor type (P = 0.345).

The headache was reported in 13 individuals with functional adenoma (29.5%) and 25 patients with non-functional adenoma (64.1%). The findings showed that headache was prevalent among patients with non-functional adenomas; therefore, there was a significant association between adenoma type and headache (P = 0.001, r = −0.346).

Adjuvant radiotherapy, as a complementary treatment, was applied for 60 patients (72.3%). Radiotherapy was also performed for 22 subjects (26.5%) after surgical failure; in addition, the mean duration of follow-up was 16 months (6-120).

Active disease was reported in only one patient (2.3%) with functional adenoma. Among patients with non-functional adenomas, five cases (12.8%) showed signs of active disease; in both groups, the disease was controlled satisfactorily (P = 0.064). In the evaluation of the relationship between tumor type and disease activity, no significant difference was observed between patients undergoing primary radiotherapy and those receiving adjuvant radiotherapy (P = 0.534).

Out of 27 patients with non-functional adenoma, 24 individuals (88.9%) had non-active disease and 3 patients (11.1%) showed signs of active disease. As to the findings, there was a significant relationship between the type of adenoma and disease control; in patients with functional adenoma, disease control was significantly higher (P = 0.004).

***Analysis of overall survival rate***

The mean follow-up duration was 30 months, with a median of 16 months (range: 6-125). We compared the overall survival rate of patients, based on the type of adenoma. Three-year survival rate was 95% in patients with functional adenomas and 84.5% in patients with non-functional adenomas. According to these results, there was no correlation between patients’ overall survival rate and adenoma type (Table 1).

**Table 1:** Relationship between type of adenoma and mean ± SD of overall survival rate

**Type of adenoma**	**Number**	**Mean ± SD**
Functional	44	31.659 ± 29.953
Non-functional	39	30.282 ± 28.799
Total	83	31.012 ± 29.246

SD: Standard deviation

## Discussion

Pituitary adenomas account for 10-15% of primary intracranial neoplasms. In the current study, we examined the clinicopathologic features of 83 patients, presenting with pituitary adenomas during 13 years. The most common signs were vision impairment and chiasm compression; also, galactorrhea was more prevalent than Cushing’s disease or acromegaly. Functional adenoma was observed in more than half of the patients. Three-year survival rate was 95% for functional adenoma and 84.5% for non-functional adenoma, although no association was found between survival rate and type of adenoma.

Rim et al.^17^ evaluated patients with pituitary adenoma, using external beam radiation therapy (EBRT). Correspondingly, it was revealed that headache, vision impairment, and hormonal disorders were the most common signs. In the mentioned study, EBRT was considered to be an effective method for controlling the pressure effects of non-functional adenoma; similarly.

Furthermore, Shao and Li^18^ in a similar study reported that headache and hormonal disorders were more prevalent among patients with pituitary adenomas; however, lower levels of hormones were observed, compared to our findings; this may be due to differences in the studied populations.

In the current study, 74.4% of the studied patients suffered from vision impairment. Another similar study performed in Egypt showed that 57% of patients had optical disorders, which is different from the findings of the present study; the mean follow-up duration was 44 months in El-Shehaby et al’s.^19^ study, while it was 31 months in our study.

In the present study, adenoma type was not significantly associated with gender, age or signs of chiasm compression, though a significant relationship was observed between adenoma type and headache severity; this could be due to hormonal activity in patients with functional adenoma. Furthermore, prolactin, IGF-1, growth hormone and ACTH levels were high in some patients in the present study.

In our study, there was a significant relationship between adenoma type and disease activity in patients undergoing complementary adjuvant radiotherapy. Becker et al.^20^ in a review study reported that non-functional adenomas are more significantly affected by radiotherapy, compared to functional adenomas (80-90%), while our findings showed that functional adenomas can be better controlled.

Rim et al.^17^ in another study showed that external radiotherapy plays a critical role in the recurrence of non-functional adenomas; correspondingly, as it is presented in this study, use of radiotherapy is recommended for controlling adenoma, although with a different impact.

Mecca et al.^21^ studied the efficacy of external conventional radiotherapy (CRT) in short- and long-term control of acromegaly; they indicated the long-term effects of CRT on active acromegaly. Similar to our study, active adenoma was controlled in several cases.

As the results indicated, 3-year survival rate was 95% for functional adenoma and 84.5% for non-functional adenoma; therefore, there was no relationship between survival rate and adenoma type.

Also, in our study, 1- and 3-year survival rates for functional adenoma were 84.6 and 23%, respectively; however, regarding the non-functional type, these values were 90.9 and 22.7%, respectively. Rim et al.^17^ reported 10-year control rates to be 96% and 66% for functional and non-functional adenomas, respectively.

We also showed that survival in functional adenoma was more than that observed in non-functional tumors. Puataweepong et al.^22^ and Wilson et al.^23^ reported the 5 years survival rate to be 91% and 87%, respectively. Zargar et al.^24^ studied the clinical and endocrine aspects of pituitary tumors. As they indicated, in an endocrine center, functional pituitary tumors were more prevalent than non-functioning tumors; similarly, we showed that functional adenomas are more frequent than the non-functional type.

***Limitations***

Unfortunately, in the present study, the exact time of disease recurrence was not recorded and many patients did not refer for follow-up sessions after the initial treatment.

## Conclusion

In the present study, type of adenoma was not associated with age, gender or signs of chiasm compression, although there was a significant association between headache and type of adenoma. Application of surgery and radiotherapy together could be a highly effective approach for treating functional adenoma, although it is less efficient for the non-functional type. It is suggested that further research with different methods be performed on larger populations to obtain better outcomes.

## Conflict of Interests

The authors declare no conflict of interest in this study.

## Notes:

**How to cite this article: **Anvari K, Kalantari MR, Samini F, Shahidsales S, Seilanian-Toussi M, Ghorbanpour Z. Assessment of clinicopathologic features in patients with pituitary adenomas in Northeast of Iran: A 13-year retrospective study. Iran J Neurol 2015; 14(4): 185-89.
